# “That’s not what was originally agreed to”: Perceptions, outcomes, and legal contextualization of non-consensual condom removal in a Canadian sample

**DOI:** 10.1371/journal.pone.0219297

**Published:** 2019-07-10

**Authors:** Konrad Czechowski, Erin Leigh Courtice, Jonathan Samosh, Jared Davies, Krystelle Shaughnessy

**Affiliations:** School of Psychology, University of Ottawa, Ottawa, Canada; Queen's University at Kingston, CANADA

## Abstract

Non-consensual condom removal (NCCR) is the removal of a condom before or during sexual intercourse without one’s partner's consent. Despite considerable news and media attention devoted to the trend (as *stealthing*), little empirical research to date has examined people’s views of the practice. The present study aimed to contribute toward generating empirical evidence to guide the discussion surrounding NCCR. We asked participants about whether or not they felt NCCR is wrong, whether there should be consequences for its perpetration, and contextualized responses within legal context. A total of 592 undergraduate students took part in an online survey inquiring about their experiences with and views of NCCR. We used descriptive statistics to determine sample prevalence and outcomes of NCCR and qualitatively analyzed responses to open-ended questions asking about perceptions of NCCR. Of participants who had engaged in penetrative sexual intercourse with a male partner using an external condom, 18.7%, 95% CI [14.4, 22.7] reported that they had NCCR perpetrated against them. The majority of these participants reported that they experienced NCCR negatively and encountered related consequences; several reported contracting an STI, experiencing an unplanned pregnancy, or both. Nearly all participants expressed that NCCR is wrong, citing reasons that included the lack of consent, possibility of unplanned or unwanted outcomes, and a betrayal of trust. In this study, we found that there was agreement that NCCR is wrong, but variability in responses regarding the circumstances under which there should be consequences for the action. These perceptions reflect the current uncertainty in law. We recommend researchers refer to the phenomenon as NCCR (rather than *stealthing*) and discuss related issues to encourage future research to adopt consistent and accurate labels and definitions for NCCR. We hope that our findings will guide future research and spur public and legal discussion on NCCR.

## Introduction

The phenomenon of non-consensual condom removal (NCCR), often referred to as “stealthing,” generated a considerable amount of attention from the popular media [1–2]. NCCR has been defined as the practice of a male removing an external male condom during sexual intercourse, without their partner’s consent [3, 4]. Some reporters have labelled NCCR as “a dangerous *new* trend” [5]; others, such as a reporter from the BBC, have described the practice as “on the rise” [6]. However, given the paucity of empirical evidence on NCCR, it is unclear if NCCR is indeed *on the rise* or a contemporary *trend*. In 2017, Brodsky contextualized NCCR within American legal context and gathered anecdotal interview information from people who had NCCR perpetrated against them [3]. Additionally, some reporters interviewed legal experts about whether or not NCCR constitutes sexual assault, at times concluding that it could [7]. Recently, researchers in Melbourne, Australia found that a third of women and a fifth of men who have sex with men (MSM) attending a sexual health centre reported ever experiencing NCCR [8]. Given the lack of empirical research on NCCR, the goal of this study was to examine perceptions of, and experiences with, NCCR in a sample of undergraduate students at a Canadian university. We also examined the Canadian legal context as it relates to NCCR, to supplement existing discussions of American and British legal contexts [3, 4].

### Non-consensual condom removal

NCCR may fit within the construct of condom use resistance (CUR). According to Davis et al. [9], CUR occurs when someone successfully resists condom use in a sexual interaction with a partner that wishes to use a condom. Some researchers have found that young men report disliking condoms for reasons including reduced physical sensation, interrupting the sexual encounter, smell, inconvenience, and financial expense [10–13]. CUR can be classified as either coercive or non-coercive. According to Davis et al., coercive CUR refers to the use of aggressive or manipulative tactics to discourage condom use (e.g., threatening to harm one’s partner if condoms are used, or asserting that condom use would make the user angry [9]; non-coercive CUR occurs when a person makes a direct request to their partner to not use the condom or does so by providing a reason such as the experience of reduced sensitivity with a condom [9]. Davis et al., found that 80% of their participants had engaged in a CUR tactic and 31% reported at least one coercive CUR tactic [9]. In sum, if men dislike condoms, some may employ various resistance tactics to avoid using them, including when their sexual partner wants to use a condom [14].

NCCR may also be considered part of pregnancy-promoting behaviours [15]; specifically, a form of *birth control sabotage*. NCCR would fit within this category of behaviours if the perpetrator’s objective was to impregnate their partner. Men who engage in birth control sabotage have done so by poking holes in condoms, expressing anger and hostility after being asked to use a condom, refusing to use a condom, throwing condoms away before attempted use, as well as removing the condom during intercourse [15, 16]. Moreover, Moore et al. [17] linked pregnancy-promoting behaviours with intimate partner violence and posited that, although the sex itself is wanted, the fact that it is without contraception makes it unwanted. Ebrahim [18] described NCCR as a distinctly masculine practice that subjugates female reproduction; though there is evidence that MSM perpetrate NCCR against other men [8].

Latimer et al., [8] examined prevalence, the context in which NCCR occurred, its impact on respondents, and how people who had NCCR perpetrated against them perceived the event. They found that a third of women and a fifth of MSM reported experiencing NCCR. They identified being a sex worker as a risk factor for women, and anxiety and depression as a risk factor for MSM. Of those who had NCCR perpetrated against them, most reported emotional distress and some reported STIs as a direct result of NCCR. When asked if they considered NCCR sexual assault, many reported they did.

### NCCR in legal context

The legal implications of NCCR are unclear at present, both in Canada–where the current study was conducted–and more broadly around the world. There are few laws or legal commentaries specific to NCCR, although articles by Brodsky [3] and Clough [4] are two notable exceptions.

Brodsky [3] provided the most comprehensive commentary on the legal complexities of NCCR to date, within the context of American law. In her article, Brodsky described interviews with people who had NCCR perpetrated against them. She described NCCR as a form of sexual violence that “puts partners at risk for unwanted pregnancies and sexually transmitted infections (STIs) and, survivors explain, it feels like a violation of trust and a denial of autonomy, not dissimilar to rape” (p. 184). Given the relevance of consent in legal considerations of sex, Brodsky contemplated how NCCR could vitiate (or nullify) consent to sex, perhaps therefore lending support to subsequent legal action. Brodsky settled on a legal preference for considering, on a fundamental literal approach to consent, that someone who has had NCCR perpetrated against them has consented to touch by a condom and not to touch by the skin of a penis. Therefore, if a condom is removed without the consent of a partner, that partner’s prior consent to sex has been vitiated. Brodsky [3] found this option preferable to the risk-based alternative to consent, which would find that NCCR vitiates consent due to a related increase in the risk of pregnancy or STI transmission. However, to our knowledge, American courts have not yet tested either of these approaches.

Brodsky [3] also described various possible legal remedies with which to approach NCCR in an American context. These included criminal law (e.g., crimes of rape or sexual abuse), tort law (involving civil legal actions between private parties, rather than between the public state and a private citizen; e.g., negligence, fraud, misrepresentation, or battery causing specific harm to the party bringing the action), contract law (e.g., violating the terms of an agreement related to sex), and civil rights law (e.g., violent crime motivated by gender). She concluded that none of these options sufficiently address NCCR and proposed a new tort for the non-consensual removal of a condom, which would require both partners’ affirmative consent to remove a condom during sex without subsequent liability.

Clough [4] considered NCCR within the context of criminal law in the United Kingdom. Primarily focused on the issue of consent, Clough contemplated:

How much you can withhold information from a sexual partner, and still claim to have reasonable belief that they are consenting to the act, without the knowledge to make an informed decision … how much information is needed for valid consent? (p. 181).

Recognizing legal uncertainty in this area, Clough concluded with the suggestion that NCCR adds more intimacy and physical contact to sex than that which all parties originally had consented. Therefore, despite evidentiary challenges in NCCR cases, Clough believed that finding a perpetrator of NCCR guilty under sexual offence criminal law is perhaps appropriate. However, to our knowledge, courts in the United Kingdom also have not yet tested the law as it relates to NCCR.

Beyond legal commentary, actual cases of NCCR have taken place in continental Europe. In Switzerland, news agency Reuters reported on a related criminal case [19]. In this reporting, Reuters described a male partner surreptitiously removing the condom despite his female partner refusing to consent to unprotected sex. A lower court found him guilty of rape, though on appeal the conviction was reduced to defilement. Thus, there was disagreement between the lower and appellate courts regarding exactly what kind of crime NCCR represents. Similarly, news agency CNN reported on a German trial court convicting a man of sexual assault (though he was originally being tried for rape) for engaging in NCCR with his partner who explicitly asked him to wear a condom [20]. In doing so, the court noted that although the sexual intercourse was consensual, the condom removal was not. However, this ruling may still be appealed, leaving some uncertainty regarding the legal context of NCCR in Germany. Ultimately, the developing law of NCCR is very much in its infancy. However, it is notable that NCCR was viewed within a criminal context and prosecuted under the criminal law in both Switzerland and Germany.

The law also remains unsettled in Canada, where the current study was conducted. For instance, there is mention of consent as it relates to sexual assault in the Canadian Criminal Code. Section 273.1(1) defines such consent as “the voluntary agreement … to engage in the sexual activity in question” (1985, p. 308 [21]). However, this vague description of consent as “voluntary agreement” has required further particularization by Canadian courts. The related case law can provide some indication of how NCCR may be treated under Canadian law.

The case of *R v Cuerrier* (1998 [22]) considered unprotected sex with a partner who knew he was HIV-positive and did not disclose this information. The Supreme Court of Canada determined (with narrow exception) that consent (i.e., voluntary agreement) to sex is vitiated by fraud when the consent is obtained through deception, the subsequent sexual interaction brings a significant risk of serious bodily harm (e.g., risk of HIV transmission), and a partner would not have consented to the sexual interaction if they had not been so deceived. This reflects the risk-based approach to consent that Brodsky [3] was disinclined to recommend–that is, a focus on the risk of negative outcomes rather than the literal components of consent. The Supreme Court of Canada also made a point of clarifying that, had there only been deception and no significant risk of serious bodily harm, this case would not be considered criminal in Canada (although they did hint that such a case might proceed via civil lawsuit).

This approach was later upheld and clarified in the case of *R v Mabior* (2012; [23]). The Supreme Court of Canada in this case maintained the risk-based *Cuerrier* approach to consent. The Supreme Court of Canada noted “the wisdom of the common law that not every deception that leads to sexual intercourse should be criminalized” (p. 607–608). At the same time, given medical advances in the intervening years, the Supreme Court of Canada clarified that a significant risk of serious bodily harm would not include the non-disclosure of HIV-positive status if viral load is low and condoms are used; these facts would negate realistic possibility of HIV transmission. The Attorney General of Canada has similarly directed prosecutors, noting that “it is not in the public interest to pursue HIV non-disclosure prosecutions for conduct that medical science shows does not pose a risk of serious harm to others” [24].

The Supreme Court of Canada again considered consent to sex in *R v Hutchinson* (2014 [25]). In this case, the man deliberately broke condoms prior to having sex with his female partner. The woman became pregnant, despite her insistence on using condoms to prevent pregnancy. The Supreme Court of Canada decided that the woman’s consent to sex was vitiated by fraud because the man was deceptive in surreptitiously sabotaging condoms, and that the resulting increased risk of pregnancy constituted risk of serious bodily harm under the *Cuerrier* approach. The Supreme Court of Canada stated:

The concept of ‘harm’ does not encompass only bodily harm in the traditional sense of that term; it includes at least the sorts of profound changes in a woman’s body–changes that may be welcomed or changes that a woman may choose not to accept–resulting from pregnancy. Depriving a woman of the choice whether to become pregnant or increasing the risk of pregnancy is equally serious… (p. 376)

These cases suggest that, in Canada, NCCR may be considered criminal only if it leads to significant risk of serious bodily harm related to STI transmission or pregnancy. Criminal liability perhaps would not be found if the NCCR only caused “financial deprivations or mere sadness or stress from being lied to” p. 377 [25].

Finally, in the Canadian civil law context, the case of *PP v DD* (2017 [26]) is notable. The Court of Appeal for Ontario considered this case about a male partner whose female partner said that she was taking the birth control pill and did not want to use condoms. However, she subsequently became pregnant and gave birth to his child. He brought civil claims against her, claiming that he suffered harm by being deprived of the opportunity to determine how and when he would start his family. He claimed that his consent to sex was vitiated because his partner misrepresented her use of contraception. The case was considered under civil law, with a less restrictive approach than the criminal law for finding vitiation of consent (as hinted in *R v Cuerrier*, 1998 [22]). However, the Court determined that the male partner did not suffer the kind of harm or violation of physical or sexual autonomy required for his consent to be vitiated in this case. In doing so, the Court also distinguished between the effects of pregnancy on men versus women, stating that “[h]is situation, as a man, is quite different from that of the woman. Clearly, there are profound physical and psychological effects on a mother undergoing a pregnancy that do not apply to the father of the child” (*PP v DD*, 2017, p. 32 [26])

This reasoning suggests that Canadian law might consider pregnancy-related differences between men and women when determining issues of consent and harm in sex-related civil suits. In the NCCR context, this could have implications for opposite-sex versus same-sex sexual interactions, or for those who can conceive a child versus those who cannot. Ultimately, whether in the United States, Europe, or Canada, the certainty of the law as it relates to NCCR appears far from settled. However, in Canada, the law favours a risk-based approach to determining consent and whether or not consent was vitiated.

### Public perspective on NCCR

Research on laypeople’s perspectives on NCCR is important for understanding social norms, current cultural values, and likely legal expectations regarding this behaviour and experience. However, we know little about the prevalence of NCCR, its outcomes, and how the public conceptualizes this behaviour and experience. Without hearing directly from laypeople about NCCR, we will be unable to develop a comprehensive understanding of this experience. Further, information on laypeople’s perspectives of NCCR is important for taking a step toward understanding the social norms and legal expectations regarding this behaviour. For instance, social scientists contributed to the national conversation on same-sex marriage in the United States prior to its national legalization in 2015, using public opinion research to inform legal and public debates on the matter [27, 28]. Although some findings suggests that many (but not all) people who reported having NCCR perpetrated against them also considered this experience sexual assault (e.g., [3, 8]), researchers have not systematically asked people about their perspectives on NCCR in an open-ended manner. We believe an exploration of people’s self-generated perspectives is essential for developing future empirical studies and informing the emerging legal and public discussions.

### The current study

NCCR is emerging as an important topic related to non-consensual sexual experiences. However, there is little empirical evidence available to guide this discussion. The purpose of the current study was to take an exploratory step towards generating empirical evidence about the prevalence and perceptions of NCCR. We examined Canadian undergraduate students’ experience with, and perceptions of, NCCR. Participants were recruited from the largest English-French bilingual university in the world, centrally located in a diverse urban location in the nation’s capital. University students are some of the most frequent condom users; they are regularly targeted in interventions designed to promote condom use [29]. Thus, their experiences with and perspectives on NCCR are important contributions to the knowledge base used to inform developments in condom use interventions (given that condoms do not work when removed). We contextualized their perceptions within the legal context in Canada, the location of the study. Previous scholars have operationalized NCCR as the removal of a condom during intercourse without the other partner’s consent. However, in the current study, we broadened our definition to include the removal of a condom *before or during* intercourse without the other partner’s consent. The addition of *before* expands the definition to be more inclusive of all NCCR experiences; limiting the definition to only *during* may miss some NCCR experiences. Moreover, we did not use the term *stealthing* because it has been used to refer to other behaviours (e.g., [30]) and because we believe non-consensual condom removal to me a more accurate, correct, and appropriate term. We also separately asked participants if they had experienced NCCR without their knowledge and consent, to examine whether and how responses may vary. We asked all participants–regardless of whether or not they had experienced NCCR–about their perceptions of NCCR, and whether they thought people should face consequences for perpetrating the behaviour. The following research questions guided this examination:

- What is the prevalence of NCCR in our sample?
- What outcomes have those who have had NCCR perpetrated against them experienced?
- Do people believe that NCCR is wrong or not? What reasons do they give for their perspective?
- Do people think there should be consequences for perpetrators of NCCR?

## Method

### Participants

A total of 671 participants completed an online survey about “perspectives on sexual behaviours.” Of these, 32 participants were identified as duplicate responders and only their first response to the survey was retained for analyses. An additional 47 participants were excluded from analyses because they either completed the survey in under 5 minutes (n = 22), responded ‘no’ to a question asking if they had answered the survey questions honestly (n = 15), or were judged to have given inaccurate or inadequate responses (e.g., left most questions unanswered and finished quickly; n = 10).

The total sample retained for analyses consisted of 592 participants. Of these, 153 (25.8%) identified as cisgender men, 435 (73.4%) as cisgender women, and 4 (0.7%) as transgender or non-binary people. Participants ranged in age from 16 to over 30 years old (11 endorsed the maximum “30+” option and were coded as 30 years old for mean and standard deviation calculations; *M* = 19.6, *SD* = 2.5, *Mode =* 18). Most participants (n = 487, 82.4%) identified as heterosexual. The majority (n = 330, 57.6%) were not in a committed relationship. Participants reported having an average of 1.9 (*SD* = 3.8) partners for penetrative sexual intercourse in the previous six months, and an average of 4.5 (*SD* = 6.9) partners for penetrative sexual intercourse in their lifetime. When asked whether they had ever experienced sexual abuse, 16.7% identified that they had. Additional sample demographic characteristics are presented in Table 1.

**Table 1:** Sample demographic characteristics.

	Subsample Size	Proportion of Total Sample in %
**Gender**		
Men	153	25.8
Women	435	73.4
Transgender and non-binary people	4	0.7
**Sexual orientation**		
Heterosexual	486	82.1
Gay	15	2.5
Lesbian	12	2
Bisexual	50	8.4
Other	28	4.7
**Relationship status**		
Committed relationship	251	42.4
Single and dating	75	12.7
Single and not dating	252	42.6
Other	13	2.2
**Types of contraception ever used (responses provided by people of all genders)**		
External male condom	324	54.7
Hormonal birth control	273	46.1
IUD	32	5.4
Morning after pill	41	6.9
Pulling out	149	25.2
None	33	5.6
Other	30	5
**Country of birth**		
Canada	476	80.4
China	22	3.7
France	6	1
Other (e.g., Ukraine, Syria, Peru)	77	13

### Procedure and measures

In 2017 and 2018, we recruited university students from the Integrated System of Participation in Research (ISPR) at a large university in Ottawa, Canada. The study was approved by the institution’s Research Ethics Board. The ISPR is an established research recruitment database that all School of Psychology researchers have access to at the University of Ottawa. Students enrolled in an introductory psychology course are able to participate in research studies for credit towards their course; they also may choose other activities for the same amount of credit should they not want to be a research participant. The ISPR database and generic recruitment system is separately approved by the institution’s Research Ethics Board. In any given term, there are several thousand students in the ISPR from multiple sections of two distinct introductory courses, both offered in English and French. None of the authors were instructors or teaching assistants of the courses from which students were recruited through the ISPR. Most students are in their first year of university; however, there also are students in upper years who take the course as an elective and people enrolled part-time, casually, or in a second degree. Once registered to the ISPR, students receive a unique identifier; this code ensures that participants remain anonymous to the study researchers (who see their code but not the identifying information connected to it). ISPR participants view a range of standardized research study descriptions and voluntarily choose which one(s) they want to participate in.

In our description, we invited participants to take part in an online survey about their “perspectives on sexual behaviours, dating habits, and dating scripts.” Participants who voluntarily registered to take part in this study gained access to a link for the survey hosted on Qualtrics. The consent provided information about the study, participants’ rights, privacy, and confidentiality including storage and limited sharing to the research group. Participants actively clicked to indicate their consent and were then directed to the online survey. The Research Ethics Board approved that university students enrolled in the ISPR who are under 18 years old may consent on their own without requiring parent or guardian consent. Participants completed background questions, questions about their sexual and relationship experiences, self-report measures of their experience with NCCR, and open-ended questions about their perspectives of NCCR. Questions about NCCR perspectives were asked in succession but were separated by a page break.

#### Background questionnaire

Participants responded to closed- and open-ended questions about their demographic characteristics (including gender, sexual identity, and relationship status), sexual experience, and whether or not they ever experienced sexual abuse.

#### NCCR experience

We used two items created for the purpose of this study to measure participants’ overall prevalence of experiencing NCCR: (i) *Have you ever had sex with a male partner who*, *during (or before) sex*, *removed the condom without your consent?* and (ii) *Have you ever had sex with a male partner who*, *during (or before) sex*, *removed the condom without your knowledge?* Response options for both of these questions were: (i) Yes, (ii) No, (iii) I have never had sex with a male partner, and (iv) Prefer not to respond. We calculated ninety-five percent binomial confidence intervals for prevalence proportions.

Participants who responded *yes* to the question about consent were presented with two follow-up questions: (i) *To what extent did this bother you?* (1 = not at all, 2 = a little bit, 3 = a lot) and (ii) *Have you experienced any unwanted or unexpected consequences as a result of this?* (Pregnancy, STI, Both pregnancy and STI, None, Prefer not to answer). Participants who responded *yes* to the question about knowledge were also asked about whether they experienced any consequences as a result of the action.

#### NCCR perspectives

We used open-ended questions to examine participants’ perspectives of NCCR. In the instructions, we encouraged participants to write “as much as [they] want” and provided a paragraph-sized text box for each answer, which allowed an unlimited amount of characters to be entered. We designed the first item to examine the extent to which participants felt NCCR was wrong, and why: *In your opinion*, *is it wrong for someone to take off the condom before or during sex without asking their partner first? Why or why not? (You can write as much as you want here)*. We intentionally chose to use “without asking” instead of the term “consent” in this item to avoid priming participants to consider consent as a possible factor when formulating their responses. We designed the second open-ended item, presented on a separate page of the survey, to examine whether or not participants felt there should be consequences for NCCR: *Should there be consequences (legal or otherwise) for [NCCR]? Why or why not? (You can write as much as you want here)*.

### Qualitative data analysis

The purpose of the qualitative data analysis was to accurately and reliably describe and summarize the content of participant responses. We aimed to derive an in-depth examination of response content that remained as close to the data as possible. We also wanted to describe how frequently types of content appeared in responses. Thus, we used first-cycle coding methods only [31]; that is, we used a simple and direct manner to create and apply descriptive codes to the data. Our aim was to describe, summarize, and essentially quantify the data to then consider how frequently certain types of responses were given. Given the nature of our open-ended electronic survey response data, we chose an approach that would aim to reliably describe what participants reported about their perspectives of NCCR. Therefore, second-cycle coding methods (the practice of reorganizing and reanalyzing first-cycle codes to organize them thematically, conceptually, and/or theoretically [31] were not adopted for this study.

All responses were imported into NVivo (version 12, Mac iOS) qualitative data management and analysis software. Written responses ranged from brief, one-word sentences to two paragraphs; most were a few sentences in length. We created separate files for men and women to allow for comparison. In doing so, we grouped the people who did not identify as cis-gender with one of the two groups. We used participants’ current reported gender identity if they identified as binary transgender (e.g., trans men with men); we used other background information to choose the gender group for participants who identified as non-binary transgender (e.g., someone identifying as heterosexual and reporting to have only had sex with women was grouped with the male participants).

Next, we developed an inductive coding structure. First, we conducted a process of Initial Coding (often referred to as "open coding" [31]) an open-ended, data-driven approach. The first author used In Vivo coding (coding with the use of unaltered text segments [31]) to extract ideas in participants' own words. In Vivo codes were used to develop an initial coding framework, which was composed of descriptive codes that were assigned to portions of data collected from each participant. Second, the second author confirmed the coding framework against the data. Third, these authors shared the codes, notes about the codes, and initial code definitions with the research team for feedback. Codes were developed to be as descriptive of the data as possible; this was feasible because the majority of respondents were clear and unambiguous with their responses. This process was repeated for both open-ended questions. In developing the coding structure for the second question, we focused on the circumstances under which participants thought that there should be consequences (instead of why there should or should not be consequences), for two reasons: (1) we found that the majority of participants focused on this aspect in their responses; (2) we observed that the answer to *why* was covered in most responses to the first open-ended question. After the coding process was complete, we merged codes that occurred infrequently. For example, we used the code “ethical/moral considerations,” for only 11 responses to the first question; thus, we merged this code with the “other reasons” code.

We took several steps to ensure the quality of these analyses. First, we maintained methodological and analytic documentation (often referred to as an “audit trail” [32, 33]), to keep track of our decision-making. Specifically, the first author maintained a detailed audit trail using a Microsoft OneNote file that the second author (and second coder) always had access to. The audit trail included details about the development of codes, notes between coders (e.g., “I’m considering merging these two codes”) and comments or questions for the larger team (e.g., “How should we deal with statements like ‘because something can happen’ without specification of that something being an STI or pregnancy? Bring up with team.” Second, the research team responsible for the analysis met regularly to discuss the development of the coding structure, the analysis, and overall impressions of the data. In our weekly meetings, we attempted to maintain reflexivity in our analysis. Specifically, we evaluated and discussed our own subjective responses to the data, with an aim at being conscious of these as best possible. [34]. Third, the second author coded a portion of the data to measure inter-coder reliability. Following the recommendations of Lombard, Snyder, and Bracken [35] we opted to use two indices of intercoder reliability: one that accounts for agreement expected by chance (Kappa statistic) and another that does not (percentage agreement; see Table 2). Fourth, we established “coding rules” [36] before beginning our analyses; these specified how codes would be applied to text (i.e., a given code applied to a participant’s full response, from start to finish, with no additional spaces) to ensure that true matches in coding were recognized.

**Table 2:** Codes for each open-ended question and their corresponding inter-coder agreement statistics.

**In your opinion, is it wrong for someone to take off the condom before or during sex without asking their partner first? Why or why not?**	**Cohen’s Kappa**	**Percentage agreement**
**Yes, it’s wrong because…**		
Lack of consent	0.85	93.45
Betrayal of trust/deception	0.92	98.69
** May result in outcome(s)**		
Sexually transmitted infections (STIs)	0.96	98.04
Unplanned pregnancy	0.97	98.69
Other/unspecified consequences	0.79	93.02
Other reasons	0.61	98.90
No reason given, just wrong	0.94	99.35
Not wrong/depends	1.00	100
Sexual violence	0.92	98.69
*Note*. Intercoder agreement calculated from 266 responses (45.31% of the data)		
**Should there be consequences (legal or otherwise) for [NCCR]? Why or why not?**	**Cohen’s Kappa**	**Percentage Agreement**
**Yes…**		
But not legal or too severe	0.89	98.95
If partner wants or pursues	0.63	97.90
If there are outcomes (e.g., STI, Pregnancy)	0.69	92.67
It’s a form of sexual violence	0.87	95.82
Just yes	0.73	86.38
No	0.89	98.95
It would be hard to prove, enforce	0.92	98.43
Unsure/ no opinion given	0.86	98.17

*Note*. Intercoder agreement calculated from 254 responses (44.63% of the data)

## Findings

### Prevalence of NCCR

Overall, 334 participants (56.4%) reported that they had, at some point in their life, engaged in penetrative sexual intercourse with a male partner and used a condom. Of these participants, 62 (18.7%; 95% CI 14.4, 22.7) reported that they had NCCR perpetrated against them at some point in their life. Specifically, 26 participants reported that they had experienced a situation in which a male partner had removed a condom during or before penetrative intercourse without their *consent*; four had experienced the same situation without their *knowledge*, and 32 reported that they had experienced this situation *both* without their consent and knowledge. Of the total 62 participants who reported NCCR, 58 identified as cisgender women, two as cisgender men, and two as transgender or non-binary people. Finally, nearly half (n = 29) of the participants who reported NCCR also reported experiencing sexual abuse when asked separately about if they had ever experienced sexual abuse (unrelated to the questions about NCCR).

### Outcomes of NCCR

On average, participants who had experienced a situation where a male partner had removed a condom during or before penetrative intercourse without their consent (n = 58) reported that the situation bothered them a great deal. Specifically, 67.2% reported they were bothered a lot by the experience, 29.3% reported they were bothered a little, and 3.4% reported they were not bothered at all. Of the 62 participants who reported having experienced NCCR, two reported that they had experienced pregnancy, four participants reported an STI, and one participant reported both as a direct consequence of NCCR.

### Perceptions of NCCR

We used two separate coding structures for each open-ended question about whether participants believed NCCR to be wrong and whether there should be consequences for its perpetration. Most responses were clear and unambiguous, which likely contributed to the high inter-coder reliability statistics displayed in Table 2. The majority of participants responded to both questions. Examples of participants’ responses for each code are reported exactly as they appeared; we did not edit or modify them in any way, including for spelling and grammar.

### Is NCCR wrong? Why or why not?

#### Yes, it’s wrong, because…

Nearly all participants reported that they thought NCCR was wrong. However, the reasons they cited for this view differed considerably. Our qualitative analysis suggested five codes represented participants’ explanations for why they believe NCCR is wrong: lack of consent, may result in outcomes, betrayal of trust/deception, no reason/just wrong, and other reasons. The proportion of the responses that were included in each of the codes are presented in Table 3.

**Table 3:** Code proportions for perceptions of NCCR.

In your opinion, is it wrong for someone to take off the condom before or during sex without asking their partner first? Why or why not?	Women (n = 432)	Men (n = 155)	Total (n = 587)
**Yes, it’s wrong because…**			
Lack of consent	61.3%	47.1%	57.6%
Betrayal of trust/deception	15.3%	7.7%	13.3%
** May result in outcome(s)**			
Sexually transmitted infections (STIs)	35%	36.1%	35.3%
Unplanned pregnancy	36.6%	24.5%	33.4%
Other/unspecified consequences	18.8%	13.6%	17.4%
Other reasons	2.1%	5.8%	3.1%
No reason given, just wrong	7.6%	11.6%	8.7%
Not wrong/depends	1.4%	0.7%	1.2%
Sexual violence	5.5%	0.4%	5.1%

*Note*. Percentages do not add up to 100 due to code overlap (i.e., multiple codes may be applied to some responses).

#### Lack of consent

Most participants specifically spoke about the lack of consent or agreement between both parties to remove the condom: “Yes, because that’s not what was originally agreed to. When the situation changes the agreement also changes.” Participants often pointed to differences between consenting to sex with or without condoms, for example: “sex with out protection is entirely different than with a condom. It has more repercussions that you are exposing your partner too with out their knowledge or consent.” By consistently drawing distinctions between sex with a condom and without a condom, participants expressed that consent to sex with a condom is separate from consent without a condom, since “there answer to having sex could change whether or not their is protection.” Ultimately, many participants emphasized the importance of partners making such decisions together: “I think the partner needs to ask because sex relates to two people. He cannot make a decision by himself.”

#### May result in outcome(s)

After consent or agreement, the possibility of undesired or unplanned outcomes as a result of unprotected sex was most commonly cited as the reason why participants felt that NCCR was wrong: “it is about avoiding STI's and unwanted pregnancy—that isn't up to just one person.” When cross-tabulating the STI and unplanned pregnancy codes, we found overlap approximately three-quarters of the time, meaning that three-quarters of participants who cited the risk of unplanned pregnancy also mentioned the risk of an STI. Often, participants cited the possibility of both outcomes together, as one man reported, “I do think it is wrong because it carries a greater risk for STI transmission (in my case) and pregnancy for heterosexual intercourse.”

In addition to specified outcomes outlined above, some participants’ responses were classified as citing *other/unspecified* unplanned or unwanted outcomes. This refers to respondents who did not specifically mention pregnancy or STI-related outcomes, but spoke of outcomes in general terms, as “different risks to consider,” or that there “might be consequences on the other partner” or broadly speaking about how each “individual is entitled to their safety” without specifying what one is entitled to be safe from. In all these instances, participants were not specific about what consequences or risks they were referring to but did suggest these as their justification for believing that NCCR was wrong.

#### Betrayal of trust/deception

A minority of participants who said NCCR was wrong cited a “breach of trust,” “a complete violation of respect and trust,” or describing it as “basically betrayal.” One participant who had not experienced NCCR stated that if she ever had it perpetrated against her, she would “have no words to describe how I would feel, other than betrayed and upset.” Participants also spoke of the *lack of respect* as an important factor that contributes to why they believe NCCR is wrong. For instance, “if the person respects you then they should respect your decision of having sex with a condom” or that “it is very wrong for someone to take off the condom before or during sex without asking their partner first since it's a complete sign of disrespect.”

#### No reason given, just wrong

We developed a code for responses that identified NCCR as being wrong but did not provide any further detail as to why NCCR is wrong. The vast majority of responses to which this code was applied were very short, simply indicating they felt it was wrong, without giving detail as to why. Examples include “yes,” “yes absolutely,” “yeah honestly I find it messed up,” or just “its wrong.”

#### Other reasons

Given the inductive nature of our method, we also identified other reasons that were raised by participants for why they believe NCCR to be wrong. However, no new codes emerged from this exercise because only 18 individuals were coded as giving *other* reasons. Some participants whose responses were coded as giving *other reasons* described NCCR in moral terms as “DEEPLY immoral,” “unethical,” or as “violating the other person's rights.” Other respondents were not entirely clear with their reasoning, such as one who wrote, “yes it's like they think they are in control of our body when they dont know ask.”

#### Not wrong or it depends

Few participants (n = 7) described NCCR as *not wrong* or wrote that *it depends* on the situation. Three of these seven individuals responded with just a “no” without providing any more details. Another three participants said they thought that it depended on whether or not the individuals involved were in a committed relationship, as in: “I think this depends on the type of relationship you are in”; “i think it depends on whom it is, and how long you've been with them”; “yes [it’s wrong] if you arnt dating and haven't had sex without a condom before.” And finally, the seventh person said they did believe NCCR was wrong, but if both partners “know their sexual health and they take other contraceptive, they don't have to use a male condom,” which we understood to mean that they thought NCCR may be acceptable under such circumstances.

#### Sexual violence

This code was applied to instances where participants specifically linked NCCR to a form of sexual violence. The label most often used by respondents was *rape*, then *sexual assault*, and then *abuse*, which was used least often. These responses tended to be longer than the others, more detailed, and more emotive. As one participant stated:

Yes, it is wrong for someone to take off the condom before or during sex without asking their partner first because it is a form of sexual assault. When someone has not explicitly and enthusiastically agreed to a sexual activity, it becomes sexual assault. When an act is not consented to, it is a violation of their partner's boundaries and expresses a perceived entitlement to their partner's body … Basically, it is wrong because it disrespects their partner's basic right to bodily autonomy

A minority of responses to which this code was applied described NCCR as something “akin to a sexual assault in my mind” or that “it could be part of a sexual abuse.” Another participant who had NCCR perpetrated against her said that:

Condom removal during sex without consent is comparable to rape … opening up your body to someone else on an intimate level is a privilege and should be treated as so. Everyone has the RIGHT to ask whatever questions they feel are necessary to feel comfortable enough to sleep with someone else.

However, the majority of these responses described the lack of consent to the removal of the condom as meriting a more unequivocal label of sexual violence. As one participant put it, “yes. It is sexual assault, because the parameters of consent have changed when the condom is removed,” another who said, “it’s called rape” and another, who simply said “this is rape because there’s no consent.”

### Should there be consequences?

Overall, 85.4% of participants wrote that they did think that there should be consequences for perpetrators of NCCR; 8.1% that there should not; and, 6.5% were unsure or gave no opinion. The proportion of responses that included each code are depicted in Table 4.

**Table 4:** Consequence code proportions.

Should there be consequences (legal or otherwise) for [NCCR]? Why or why not?	Women (n = 419)	Men (n = 150)	Total (N = 569)
**Yes, there should be consequences**			
But not legal or too severe	6.7%	5.3%	6.3%
If partner wants or pursues	4.3%	4.7%	4.4%
If there are outcomes (e.g., STI, Pregnancy)	11.2%	11.3%	11.3%
It’s a form of sexual violence	14.8%	11.3%	13.9%
Just yes	51.1%	49.3%	50.6%
No	6.9%	11.3%	8.1%
It would be hard to prove, enforce	8.1%	6.7%	7.8%
Unsure/ no opinion given	6.4%	6.7%	6.5%

*Note*. Percentages do not add up to 100 due to code overlap (i.e., multiple codes may be applied to some responses)

#### Yes, there should be consequences

Most participants reported that there should be consequences for perpetrating NCCR. The majority either wrote only an agreement response. However, many participants qualified their answer with an “if” or a “but” that placed an emphasis on the consequences or outcomes of the experience.

#### Just yes

Many participants simply said “yes” or “absolutely” without elaborating further. However, most followed their “yes” with an elaboration as to why, reflecting the reasons they gave for whether or not they think NCCR is wrong in their response to the previous open-ended question: “yes there should be, because it is not right, the girl could get pregnant and be put in a situation she doesn't want to be in.” Most participants were unequivocal in their response, such as one woman who had not experienced NCCR and wrote, “yes, the partner needs to understand what he did is not right” or another who was even more straightforward, “yes jail time.”

#### If partner wants or pursues

A number of respondents reported that “the victim should have options if incase he/she wishes to pursue them.” Some explicitly spoke of the legal context where “the ‘victim’ should be able to press charges” or to “sue the person because the other partner didn't want to have sex without a condom and it was clear at first.” One qualified that she “believe it shouldn't be automatic legal consequences, but the partner who has been wronged should have that option as well as resources to make other decisions.” Ultimately, many felt that those who had NCCR perpetrated against them “deserve the right to seek compensation/for the perpetrator to receive legal consequences for their crime.” A minority of participants added there should be no consequences if none are desired: “there should be consequences if the partner wants to do something about it but if they didn't feel like it was a big deal then no punishment would be needed” or as another further clarified: “it becomes a quarrel between the people involved in the relationship.”

#### If there are outcomes

Many participants felt there should be consequences for NCCR only “if it leads to STIs, pregnancy or has other consequences.” Generally, participants spoke of physical outcomes such as if “an STI or a pregnancy was to occur” or “if impregnated there should be some law that would depict a punishment for the condom user to face.” In some cases, participants further elaborated as to why outcomes merit consequences: “if the women gets pregnant, i believe there should be consequences because she must now deal with the process of either an abortion or to keep the child.” Some spoke in more general terms, not specifying the outcome they were referring to: “yes, if there was a negative outcome as a result of this action the person being deceived should be able to attain justice.” Finally, some explicitly said they felt there should be no consequences for NCCR if there are no outcomes, as one participant wrote, “not unless it results in pregnancy, but otherwise [consequences] not necessary.”

#### It’s a form of sexual violence

When stating there should be consequences for NCCR, many respondents linked NCCR directly to sexual violence, stating that “[NCCR] is sexual assault”; “yes because it is rape”; “sexual harassment/ assault”; “endangering your partner and rape”; “abusive misconduct”; or simply, “I believe that is abuse.” A minority of these respondents were more ambivalent in their responses, stating that “it could be treated a sexual abuse,” or that it may be “similar to sexual assault,” or as one participant wrote, “in some cases it could become sexual assault.” However, most participants, were unambiguous in their description; as one participant responded, “das rape son.”

#### No

A minority of the sample indicated there should be no consequences for NCCR. Some simply said “no” without elaborating further, and many commented on the legal aspect, that “there should not be legal consequences” or even that “its not a crime” (presumably implying that therefore there ought not to be a consequence). Some commented on the feasibility of prosecution, that “it would be to messy if there was legal consequences, there would be no concrete evidence” or even that it “would be near impossible to prove and could lead to false allegations.”

Some participants seemed to shift some responsibility from the perpetrator to also include the person who had NCCR perpetrated against them. One woman who had not experienced NCCR said, “no I don't think there should be consequences, because I don't think that it is a big enough issue and I think that it is more appropriate to be kept quiet and between the individuals involved” while another said she doesn’t “believe there should be legal consequences on this matter. I believe that two partners must, above all, communicate before engaging in sexual intercourse” and a male respondent echoed the sentiment, expressing that “because it is not a crime. If the person in question did something you didn't like then don't have sex with them.” Finally, some participants felt that, although it may be wrong, there should not be any consequences “because its not a crime for being a dick” or quite simply, “i dont think so but i dont think its okay to do [NCCR].”

#### Unsure, unclear, no opinion given

Some respondents simply said they “don’t know” or wrote “no comment.” One qualified his response by stating he’s not “informed enough about any of this,” another said he is “not in a position to state what [the consequences] should be. Not knowledgeable enough in the field” or even that “this should be determined by lawyers, politicians and judges.” Some respondents simply commented on the matter of NCCR, without giving their view regarding consequences. For instance, one who said she “personally [thinks] it would be hard to get consequences because there is little proof, words against each other” while another ambiguously commented that she doesn’t know since “this is a tricky situation. Sometimes people can use that in their advantage to ruin someones life or it can also save someone's life.”

#### It would be hard to prove, enforce

Some participants qualified their responses by adding that it would be “hard to prove whether or not the removal was consensual or not” or “hard to get consequences because there is little proof.” Some felt it “would be hard to tell whether the accusation is legitimate” and ultimately, consequences “would be difficult to enforce.” Responses indicating that there should be consequences were often contextualized against what many perceived as difficulty in proving legal culpability, that “it would become complicated categorizing what is and isn't legal in certain situations” and “it would be very challenging to have evidence and make a case for or against. I wish there were an easy way to do things.” Participants often qualified their “yes there should be” response with “but it would end up in a he said—she said debate,” underscoring the difficulty they perceived in prosecuting NCCR. This was illustrated by one participant who said, “it would be difficult to enforce as it would always be one person's word against the other's.”

### Gender differences in participant perceptions

As described in the method section, we split the data file by male-identified (men) and female-identified (women) when we coded for whether or not participants thought NCCR was wrong and if there should be consequences. We examined the proportional frequency of each code for each question by gender. The proportion of male- and female-identified participants whose response included each code is reported in Tables 3 and 4. Overall, we observed that our male and female participants held similar beliefs that NCCR was wrong, their responses reflected similar reasons for it being wrong, and they reported similar content regarding whether or not there should be consequences. We also observed that women gave more reasons to support their perspective that NCCR was wrong than did men. Although we did not test for gender differences in responses to the open-ended questions statistically (given the nature of our qualitative study design, we did not believe it appropriate to do so), we observed that the largest difference between the men and women in the proportion of responses including a code occurred for considering NCCR as *sexual violence* (5.5% of women; 0.4% of men) and *betrayal of trust/deception* (15.3% women; 7.7% men). When asked about whether or not there should be consequences for NCCR, responses were relatively similar between men and women.

## Discussion

The purpose of this study was to contribute towards a small but growing body of empirical evidence about the prevalence, outcomes, and people’s perceptions of NCCR. Specifically, we examined young people’s experience with NCCR, the outcomes of those experiences, whether they perceived NCCR as wrong, and what (if any) consequences they thought perpetrators of NCCR should face. We found that 18.7% of participants who had ever engaged in penetrative sexual intercourse with a male partner and used a condom reported that NCCR had been perpetrated against them. The prevalence among our sample is lower than previous research (who operationalized NCCR in a slightly different manner, as we review in the next section) that estimated prevalence rates at 32% for women and 19% for MSM [8]. On average, these participants reported that the situation “bothered” them “a lot.” Moreover, seven of those who had experienced NCCR reported they also experienced outcomes such as pregnancy and/or an STI as a result of NCCR. These outcomes are similar to those reported by Latimer at al’s participants, of whom most reported emotional distress and some reported STIs as a consequence of NCCR. [8].

The vast majority of participants indicated that they thought NCCR was wrong. Participants identified a number of reasons why they believed NCCR was wrong including the lack of consent, betrayal of trust, possibility of STIs or pregnancy, or because they considered it a form of sexual violence. Participants generally indicated that they thought perpetrators of NCCR should face consequences for the act; although some specified that consequences would be warranted only if the receiver of NCCR wanted to pursue them or if the NCCR caused unwanted outcomes. These diverse perceptions aligned with the unclear legal context of NCCR in Canada.

### NCCR research

To assess experiences, we asked if participants had ever experienced condom removal without their (i) *consent* and (ii) *knowledge* separately. We found that only four participants reported that they had experienced condom removal without their *knowledge* (but not without their consent); the rest reported their experiences either as without consent or without consent *and* without knowledge. These findings support the validity of focusing on consent over knowledge in descriptions of NCCR consistent previous definitions of NCCR as an act occurring without *consent* [3, 4, 8]. We recommend that future research describe NCCR as condom removal without *consent* and not as condom removal without *knowledge*.

However, these results also suggest that some people may experience condom removal without their knowledge but not consider the experience “non-consensual.” There are at least two possible explanations for this finding. First, it is possible that some people may rationalize condom removal without knowledge as something that was not necessarily non-consensual–perhaps if consent was simply left unaddressed. Second, some people may experience condom removal without their knowledge, however consent may be correctly implied by the person removing the condom. For instance, the condom remover may have been the only person of the pair who wanted to use a condom in the first place; whereas, the person who had the condom removal perpetrated against them was either indifferent to condom use or expressed preference to have sex without a condom. Thus, in some instances, condom removal may occur without knowledge, yet not exactly be perceived as without consent; future research in this context considering direct versus implied consent may be helpful in further examination of NCCR.

In this study, we used the term NCCR instead of *stealthing*. *Stealthing* is a term popularized by media that does not reflect the non-consensual nature of the act. Some have argued that the term *stealthing* has given people who had experienced NCCR a vocabulary to describe and talk about their experience (e.g., [18]). Others have acknowledged that people who had NCCR perpetrated against them struggle to name what happened to them (e.g., [3]). We believe that non-consensual condom removal (or “NCCR”, for those who prefer acronyms) is a more descriptive, accurate, and correct label for the phenomenon and that it has the same utility of giving people a vocabulary to describe and discuss NCCR experiences as the term *stealthing*. First and foremost, NCCR reflects the non-consensual (thus problematic) nature of the act. Second, this fairly specific term likely has less risk of sounding *trendy*. Additionally, *stealthing* is a term that is less descriptive than NCCR, and more open to (mis)interpretation. For instance, it has previously been used in other ways such as to refer to the practice of HIV-positive men intentionally transmitting HIV to HIV-negative men by engaging in unprotected penetrative intercourse [30]. Furthermore, researchers have clearly demonstrated that participants interpret popular terms used in sex research in a wide variety of ways, some of which may or may not fit with the behaviours under investigation (e.g., [37, 38]).

We also expanded the definition of NCCR to include instances of removal of the condom prior to (in addition to during) penetrative sexual intercourse. Previous scholars defined NCCR as occurring *during* penetrative sexual intercourse [3, 4, 8]. Given that the essential component of NCCR is the *removal* of a condom without consent, and not the timing of the removal, we believe that limiting the definition to only *during* penetrative sexual intercourse is not sufficiently sensitive to capture all NCCR experiences. Latimer et al., [8] also considered the bounds of NCCR by focusing on whether or not sex continued after condom removal. Specifically, they operationalized NCCR in the following way:

Participants were deemed not to have experienced [NCCR] if they responded either: 1) they had never had a condom removed during sex, 2) that a condom had been removed with permission, or 3) that a condom was removed without permission but they willingly continued sex. Participants were deemed to have experienced [NCCR] if they reported: 4) condom removal without permission and sex continued unwillingly, 5) condom removal without permission and sex was discontinued, 6) condom removal during sex but they did not realise until afterwards, or 7) the condom was never put on despite being requested. (p. 3)

We appreciate Latimer’s consideration of whether sex continued after condom removal; future researchers should continue to examine this. However, we believe their definition of NCCR excludes some behaviours that should be considered as part of NCCR and at the same time is too inclusive of some experiences that may not technically constitute NCCR. For instance, we believe their definition is too narrow because they exclude situations where a condom was removed without consent (regarding point three of their operationalization). Although the continued sex may be consensual, the *condom removal* is *non-consensual;* thus, fitting within the definition of NCCR. We believe that whether or not people continue sexual intercourse willingly or unwillingly is separate from NCCR. At the same time, (regarding point seven of their operationalization) if a condom was never put on in the first place, it cannot be removed and thus would not constitute *condom removal*. In sum, we recommend that researchers refer to the act as non-consensual condom removal (NCCR). We also recommend that a common operational definition of NCCR include experiences of condom removal *before or during* penetrative sexual intercourse without one’s partner’s *consent* regardless of sex continuing willingly or unwillingly.

Our findings suggest that research on NCCR fits with research on condom use resistance (CUR [9]), birth control sabotage [15], or more broadly, sexual violence. NCCR was no doubt a successful method for CUR for those who had perpetrated NCCR against some participants in our sample. A third of our participants explicitly said that NCCR was wrong because it could result in an unplanned pregnancy, suggesting this was an important risk they were mindful of when discussing NCCR. However, classification of NCCR as birth control sabotage largely depends on the motivations of the perpetrator of NCCR, specifically, whether they engaged in the practice to increase the chance of impregnating their sexual partner.

Insofar as NCCR is a form of birth control sabotage, it may also be considered a form of sexual violence. Indeed, previous literature has linked birth control sabotage to intimate partner violence and domestic violence [15, 16]. Further, the lack of consent inherent to NCCR also suggests that conceptualizing it as a form of sexual violence may be appropriate–this is consistent with the perceptions of some of our participants who explicitly labelled it as a form of sexual violence, and previous research that found a majority of respondents viewed NCCR as sexual assault [8].

Previous literature has suggested NCCR may be a form of gender-based violence; an act influenced by gendered motivations such as men having a right to women’s bodies, thus NCCR being their right [3]. Moreover, Ebrahim conceptualized the construct of NCCR “as a practice of hegemonic masculine dominance over female sexuality and reproduction,” (p. 5) a distinctive form of gender-based violence [18]. Although we found evidence that NCCR was viewed as a form of sexual violence by some, we found little evidence that our participants viewed it as specifically a form of gender-based violence (i.e., a behaviour directed at women because of their gender, shaped by their gender roles and status in society [39, 40]). Ebrahim, along with Brodsky, relied on online accounts from perpetrators of NCCR to inform their examination of the issue [3, 18]. Ebrahim reported that “a reading of the online narratives indicates, first, that [NCCR] is a distinctly masculine practice intended on subjugating the sexual and reproductive rights of women” (p. 6). Some of our participants who had NCCR perpetrated against them were cisgender men and Latimer et al. found that a fifth of their sample of MSM reported having NCCR perpetrated against them [8]. Therefore, NCCR is not distinctly a masculine practice perpetrated against women; it is sometimes perpetrated against men. Moreover, to our knowledge, there currently is no available evidence on whether women perpetrate NCCR against men. We strongly encourage future researchers to study perpetrator motivations and characteristics since views based on online reports of alleged perpetrators mat not be representative or typical of people who perpetrate NCCR. In the absence of empirical evidence, speculating on motivations may run the risk of inaccurately understanding the nature and potential complexity of NCCR, which may impede prevention efforts. Given the various conceptual possibilities, empirical research examining partner dynamics and perpetrator motives is needed to clarify from which theoretical framework(s) NCCR is best conceptualized and to inform prevention efforts.

### Legal contextualization of NCCR

Although almost all respondents reported believing that NCCR is wrong, their reported reasons for believing so varied. Similar to the Supreme Court of Canada’s approach to consent, some respondents focused on how the risks inherent in NCCR make the practice wrong. The Supreme Court of Canada has favoured a risk-based approach to consent and its vitiation (e.g., *R v Cuerrier*, 1998 [22]). The risk-based approach would find that NCCR vitiates consent because of a related increase in the risk of pregnancy or STI transmission. Consistent with this approach, approximately one-third of respondents explicitly raised risk of STI transmission and one-third of respondents explicitly raised risk of pregnancy as reflecting what is wrong with NCCR. It was less clear whether any degree of STI transmission or pregnancy risk would suffice or whether there was a specific *degree* of such risk that made NCCR wrong (although some did mention pregnancy risk may be mitigated by another form of contraception).

In contrast to the risk-based approach, approximately two-thirds of our respondents favoured a more literal approach to consent. This literal approach is consistent with Brodsky’s conclusions [3]. The literal approach to consent would find that someone who has had NCCR perpetrated against them has consented to touch by a condom and not to touch by the skin of a penis. Some of the individuals that Brodsky interviewed raised concerns about NCCR related to its violation of bodily autonomy, violation of trust, violation of sexual agreement, and disempowerment. Respondents in this study also raised these kinds of concerns about why NCCR is wrong; respondents seemed focused on both the lack of affirmative and explicit consent to removal of the condom and the betrayal of trust that this action accompanies. In sum, our respondents viewed NCCR through both risk-based and literal approaches to consent.

As described by Brodsky [3], each of these two approaches to consent come with different legal and policy implications and challenges. For instance, the risk-based approach requires a determination of just how much risk is required to vitiate consent. Will we be required to disclose fertility and STI status to all partners prior to sex to ensure that all possible risks are explicitly known? Alternatively, the literal approach requires drawing a line between agreed upon and non-agreed upon sexual activity. However, where exactly to draw such a line is unclear. As Brodsky [3] asked: would we accept the argument that someone who consents to touch of their left breast does not also consent to touch of their right breast? These are some of the questions that any policymaker would face when deciding which approach to consent to pursue (for further discussion on risk versus literal approaches see Brodsky [3]).

Aside from whether NCCR is wrong and why, there is also the issue of how to remedy NCCR under the law or otherwise, if at all. The majority of respondents felt that some kind of consequence should be applied to NCCR perpetrators, although they were often unclear about what kind of consequences. Respondents in the current study sometimes recommended consequences for NCCR in the context of criminal conviction and jail time for the perpetration of sexual assault. Brodsky’s [3] interviewees reportedly tended to identify NCCR as similar, but not equivalent, to sexual assault. Latimer et al. reported that most respondents agreed that sexual assault is an appropriate label, although they did not generate this label on their own [8]. In our sample, participants tended to be more explicit in labelling NCCR as sexual assault (rather than as similar to sexual assault as reported by Brodsky’s interviewees [3]). Regarding terminology, respondents most often used rape, then sexual assault, and then abuse. This suggests that when they did refer to sexual violence, they most often used the more narrowly defined label of *rape* (typically defined legally in the United States—and perhaps colloquially in Canada—as sexual penetration obtained by force [41]) rather than the more broadly defined term of sexual assault, (which typically includes rape in addition to unwanted touching [41]). Additionally, we found that nearly half of participants who had NCCR perpetrated against them answered *no* when asked about whether or not they had ever been sexually abused. It is unclear how each respondent understood the term *sexual abuse* and we cannot assume that those who did report that they had experienced sexual abuse were referring specifically to the NCCR they experienced. However, this finding does suggest that, for nearly half of participants, their experience with NCCR may not have come to mind when asked about experiences involving sexual abuse.

Some respondents also considered civil law remedies for NCCR, notably referring to monetary compensation. Respondents particularly implied that these consequences should follow from the negative outcomes (e.g. pregnancy, STI transmission) that NCCR can create. They also sometimes noted that NCCR should be dealt with in this way because it is a private issue between two individuals (a more classically civil law conceptualization) rather than between the state and individuals (a more classically criminal law conceptualization).

Conversely, some respondents said that NCCR is not criminal and no legal consequences should follow from it. Some also added that, while no legal consequences should be involved, it is still wrong and there could be other consequences involved (e.g., within the private context of a relationship). A smaller number of respondents believed that no consequences should follow from NCCR, especially if the NCCR did not lead to negative outcomes such as STI transmission or pregnancy. This approach requiring actual *realization* of negative outcomes is more restrictive than the current Canadian legal context which looks for a particular *risk* of negative outcomes, whether or not they are realized.

Regardless of how NCCR is conceptualized in legal context, some participants described challenges in proceeding with cases of NCCR in the real world. Specifically, respondents were concerned that NCCR cases would reflect a “he said she said” scenario of one person’s word against another’s. This could reflect one piece of the broader ongoing debate regarding challenges in the reporting, prosecution, conviction, and incarceration of sexual assault (e.g., [42]). Therefore, beyond conceptual considerations of the legality and consequences of NCCR, more practical issues exist: How to prove NCCR in legal proceedings and how to determine the appropriate consequence to apply.

### Differences in perceptions

We observed a few descriptive differences between our subsamples of women and men within our qualitative analysis. First, women tended to give more detailed justifications for their positions and responses than men did. For instance, when providing reasons for why they felt NCCR is wrong, women provided more reasons than men. Similarly, the proportion of women who provided a justification for their views was higher compared to the proportion of men. Moreover, women (compared to men) described more concerns about unplanned pregnancies, and the betrayal of trust associated with NCCR. Second, more men indicated that there should not be consequences for NCCR compared to women. This finding may be because more women than men explicitly linked NCCR to sexual violence. Considering the qualitative nature of our data, we did not statistically test for these gender differences. Despite our high inter-coder reliability, our findings may not represent significant differences. Indeed, most participants, regardless of gender, thought that there should be consequences for NCCR. Previous research found that very similar proportions of men and women considered NCCR sexual assault [8] but more research with more diverse samples is needed for greater confidence in gender comparisons.

### Limitations and future research

This study is not without limitations. First, given the relatively young age of our sample (mean age = 19.8), we expect that our results may be an underestimation of the overall prevalence of NCCR. Indeed, considering that many of our participants will continue to engage in penetrative intercourse, those who had not experienced NCCR at the time of this study may be exposed to situations in which NCCR may occur in the future. Thus, larger and more age-diverse samples are needed for a more accurate estimation of the prevalence and outcomes of NCCR. Furthermore, the extent to which the findings generalize to a more diverse, non-Canadian, non-urban, population is unclear. We employed a convenience sampling strategy of students who took a psychology course and agreed to participate in English research. The views of these participants may differ slightly than students who did not take a psychology course. Further, participants chose our study from a list of dozens of other studies, most of which did not advertise as studies that will inquire about sexual intimacy and relationships. Given the evidence that more positive sexual attitudes and greater sexual experience are predictors of willingness to volunteer for sexuality research [43], it is possible our participants disproportionately share those characteristics and others introduced by selection bias.

Second, we relied on a self-report survey focused on the prevalence, perceptions, and outcomes of NCCR. Thus, our findings are limited to a few variables that were our focus at the time of the study. Specifically, we decided that it was important to first focus on those who had NCCR perpetrated against them. We only examined if people had ever experienced NCCR, not how many times people had an NCCR experience, nor the partner contexts of these experiences. The former information is important to better understand whether some people are at greater risk for recurring NCCR. The latter is important to determine whether NCCR may be more or less common within romantic relationships, or, alternatively, with casual sex partners. We also did not ask whether people had perpetrated NCCR. Thus, we may unknowingly have perpetrators of NCCR in our sample. If so, their inclusion may have contributed to qualitative codes about NCCR not being wrong, or to the *it depends* codes resulting from the question about whether there should be consequences for NCCR. This information is important to ensure that the research captures the range of perspectives that exist in the population. However, more information is needed. In studying the experience of NCCR perpetration, it may also be useful to measure motivations for NCCR. Measuring motivations is essential to develop an accurate understanding of why people perpetrate NCCR, which would be an important step toward generating effective prevention strategies. Together, this additional information will be particularly important for considering policy level or sexual health education recommendations.

## Conclusion

This study was one of the first empirical investigations of prevalence, outcomes, and perspectives of NCCR. As such, despite some limitations, our findings substantially contribute to research and knowledge of NCCR. We found that a sizeable minority of our student participants who have had sex with men and used condoms have also had NCCR perpetrated against them. Given the characteristics of our sample (e.g., lack of age diversity), it is likely that the lifetime prevalence in the general population is higher. The vast majority of participants thought that NCCR was wrong and that there should be consequences for its perpetration. However, perspectives on why consequences should exist, what these consequences ought to be, under what circumstances they should occur, and how to legally proceed varied. Some participants appeared to favour a more literal approach to consent, while others preferred a risk-based approach. These varied perspectives perhaps reflect the limited presence of NCCR in our cultural awareness, as well as the untested and uncertain related legal context in Canada and around much of the rest of the world. As a result, our findings are important for informing public discussion, education, and policy initiatives as well as spurring future research on this phenomenon. We expect our study will be followed by many subsequent investigations of non-consensual condom removal.
